# A new pathway of glucocorticoid action for asthma treatment through the regulation of PTEN expression

**DOI:** 10.1186/1465-9921-12-47

**Published:** 2011-04-14

**Authors:** ZhenHua Ni, JiHong Tang, ZhuYing Cai, Wei Yang, Lei Zhang, Qingge Chen, Long Zhang, XiongBiao Wang

**Affiliations:** 1 Department of Respiratory Medicine, Putuo Hospital, Shanghai University of Traditional Chinese Medicine, Shanghai, 200062, PR China

## Abstract

**Background:**

"Phosphatase and tensin homolog deleted on chromosome 10" (PTEN) is mostly considered to be a cancer-related gene, and has been suggested to be a new pathway of pathogenesis of asthma. The purpose of this study was to investigate the effects of the glucocorticoid, dexamethasone, on PTEN regulation.

**Methods:**

OVA-challenged mice were used as an asthma model to investigate the effect of dexamethasone on PTEN regulation. Immunohistochemistry was used to detect expression levels of PTEN protein in lung tissues. The human A549 cell line was used to explore the possible mechanism of action of dexamethasone on human PTEN regulation *in vitro*. A luciferase reporter construct under the control of PTEN promoter was used to confirm transcriptional regulation in response to dexamethasone.

**Results:**

PTEN protein was found to be expressed at low levels in lung tissues in asthmatic mice; but the expression was restored after treatment with dexamethasone. In A549 cells, human PTEN was up-regulated by dexamethasone treatment. The promoter-reporter construct confirmed that dexamethasone could regulate human PTEN transcription. Treatment with the histone deacetylase inhibitor, TSA, could increase PTEN expression in A549 cells, while inhibition of histone acetylase (HAT) by anacardic acid attenuated dexamethasone-induced PTEN expression.

**Conclusions:**

Based on the data a new mechanism is proposed where glucocorticoids treat asthma partly through up-regulation of PTEN expression. The *in vitro *studies also suggest that the PTEN pathway may be involved in human asthma.

## Background

Bronchial asthma is a chronic inflammatory disorder of the airways, with episodic occurrences of airflow obstruction, and hypersensitivity and hyperresponsiveness to various stimuli. Asthma is one of the most common diseases, occurring in approximately 300 million people of all ages and ethnic backgrounds worldwide [1,2]. Many attempts have been made over decades to discover the etiology of the disease, and thousands of papers have been published. Although the mechanism is still not well understood, inflammation has been identified as the main reason that could explain most of the symptoms of asthma [3,4].

Because the dominant pathological feature is airway inflammation, one of the main achievements of the last decade has been the understanding of the inflammation nature of the disease [4,5]. In view of the unclear etiology of asthma, the purpose of asthma treatment is to achieve and maintain clinical control. Although the guidelines for asthma management by the Global Initiative for Asthma (GINA) have gone through many revisions since 1989 [6], the status of corticosteroids in this management has been stable because of their most effective anti-inflammatory function. Inhaled glucocorticoids are the most effective control currently available. Systemic administration of glucocorticoids are commonly used in the treatment of severe acute exacerbations because they prevent the progression of asthma exacerbation, reduce the need for referral to emergency departments and hospitalization, prevent early relapse after emergency treatment, and reduce the morbidity of the illness. The mechanism of glucocorticoids in asthma therapy has been explored for decades. Genomic and non-genomic mechanisms have been reviewed recently by Alangari [7], and more efforts are still being made to further our understanding of the mechanisms to help with application to therapeutics.

The PTEN (Phosphatase and tensin homolog deleted on chromosome 10) gene has been identified as one of the most commonly lost or mutated tumor suppressor genes in humans. It functions as a plasma-membrane lipid phosphatase that antagonizes the PI3K (phosphoinositide 3 kinase)-AKT pathway. PTEN exerts a wide range of effects on cell growth, migration, death, and differentiation[8]. The gene has drawn interest concerning its potential role in asthma in recent years. It has been confirmed that PTEN expression is down-regulated in an asthma model, and that exogenous PTEN can effectively relieve asthma in these mice [9-11], and reduce chronic airway inflammation and airway remodeling through regulation of IL-17 expression [12]. Administration of peroxisome proliferator-activated receptor gamma (PPARgamma) agonists or AdPPARgamma reduced bronchial inflammation and airway hyperresponsiveness by up-regulating PTEN expression in allergen-induced asthmatic lungs [13]. It has been found recently that PTEN can inhibit human airway smooth muscle cell migration [14] as well as endothelial nitric oxide synthase [15], which, in turn, inhibit airway inflammation. Because of these facts, PTEN has been proposed as a therapeutic target for asthma [16]. PTEN acts as the catalytic antagonist of PI3K by dephosphorylating PIP3 to PIP2. PI3Kbeta, delta and gamma isoform-specific PI3K inhibitors (TGX-221, IC87114 and AS-605240) have been developed for asthma treatment [17].

The evidence for the involvement of PTEN in asthma in humans, however, is rare. Moreover, there are no available data on the effects of glucocorticoids on PTEN expression. In this study, we discovered that dexamethasone could upregulate PTEN expression in mice and in a human lung epithelial cell line. We also describe a new signaling pathway for steroids in asthma.

## Methods

### OVA-induced mouse model of asthma

All experimental procedures conformed to international standards of animal welfare, and were approved by the Institute Animal Care and Use Committee of Shanghai University of Traditional Chinese Medicine. Female BALB/c mice were purchased from Shanghai SLAC Laboratory Animal Co. Ltd. All mice were kept in well-controlled animal housing facilities, and had free access to tap water and food pellets throughout the experimental period. Female, 6-8-week-old BALB/c mice (*n *= 30) were divided into three groups: OVA-treated group (OVA-challenged mice treated with saline), OVA+dexamethasone-treated group (OVA-challenged mice treated with dexamethasone) and a saline-group (saline-challenged mice treated with saline). Mice were challenged with Ovalbumin (OVA) (Grade V; Sigma Aldrich, Shanghai, China) by intraperitoneal and intranasal routes. OVA treated (*n *= 10) and dexamethasone treated (OVA/DM) (*n *= 10) groups were immunized by intraperitoneal (i.p.) injections of 100 μg of OVA mixed with potassium aluminum sulfate on days 0 and 14 [18]. Mice received an intranasal dose of 500 μg OVA on days 14, 25, 26, 27. The control group (*n *= 10) received normal saline with alum i.p. on days 0 and 14 and normal saline without alum intranasally on days 14, 25, 26, 27 [19]. The group of dexamethasone-treated mice was administered with dexamethasone intraperitoneally (1.7 mg/kg, once a day) beginning on day 28 of the protocol and continuing until day 41. Animals were sacrificed by i.p. injection of pentobarbital at day 42, and the lungs and extrahilar tracheobronchial airways were rapidly dissected out.

### Tissue processing and immunohistochemistry analysis

Immunohistochemistry detection of PTEN was done as described elsewhere [9]. Tissue sections from the right lungs were first treated with PTEN antibody (R&D Systems, Minneapolis, MN, USA). After incubation at 4°C overnight, tissue sections were washed with PBS, and treated with ligation enhancing buffer (Maixin Bio, FuZhou, China)) for 30 min at room temperature. Tissue sections were then washed with PBS, and treated for 30 min with horseradish peroxidase (HRP)-anti-rabbit IgG (Maixin Bio). The color was developed using diaminobenzidine (DAB). The intensity of PTEN protein staining was determined as an average optical density by IPP software (Image-Pro Plus 6.0, Media, Cybernetics). A non-stained region was selected and set as the background.

### Cell culture

The lung epithelial cell line, A549, was purchased from the Institute of Cell Biology (Shanghai, China), and cultured in RMPI1640 medium (Gibco, Shanghai, China) supplemented with 10% fetal bovine serum, penicillin and streptomycin. A549 cells were treated with the indicated concentrations of dexamethasone for 24 h. Otherwise, the cells were treated with 1 × 10^-5 ^M dexamethasone. The cells were harvested at 24 h, 48 h, 72 h, and 96 h.

### PTEN expression analysis by real-time quantitative PCR

Total RNA from A549 cells were extracted by Trizol (Invitrogen Life Technologics, Carlsbad, CA, USA). The RNA (0.5 μg) was reverse transcribed to cDNA, using a RevertAid First Strand cDNA Synthesis Kit (Fermentas, ShenZhen, China). Quantitative real-time PCR was performed by Universal Master Mixer (Roche Applied Science, Shanghai, China) on a 7300 Real-time PCR System (Applied Biosystems, Foster City, CA, USA). The primers and probes used are listed in Table 1. Each assay was performed in triplicate. The PCR conditions used in all reactions were: 10 min at 95°C, followed by 40 two-step cycles (95°C for 15 s and 60°C for 45 s). The relative expression levels of the PTEN gene were normalized against GAPDH and analyzed by the 2^-ΔΔCt ^method [ΔΔCt = (Ct _PTEN _- Ct _GAPDH _)_sample _- (Ct _PTEN _- Ct _GAPDH _)_control_].

**Table 1:** Sequences of primers and probes

Primers or Probes	Sequence
Forward Primer (PTEN)	5'-GGGACGAACTGGTGTAATGATATG-3'
Reverse Primer (PTEN)	5'-ATAGCGCCTCTGACTGGGAATAG-3'
TaqMan Probe (PTEN)	5'-fam- CCCTTTTTGTCTCTGGTCCTTACTTCCCC -tamra-3'
Forward Primer (GADPH)	5'-CCACTCCTCCACCTTTGAC-3'
Reverse Primer (GADPH)	5'-ACCCTGTTGCTGTAGCCA-3'
TaqMan Probe (GADPH)	5'-fam- TTGCCCTCAACGACCACTTTGTC -tamra-3'

PTEN promoter-F	5'-GGGGTACCGTGTATCCTTCCACCTCC-3'

PTEN promoter-R	5'-GAAGATCTGGCCTCGCCTCACAGCGGCTCAACTC-3'

### Reporter construct, transient transfections and luciferase assays

The PTEN promoter sequence was amplified from human blood cells. Primers were designed according to human genomic PTEN (GenBank accession no. AF067844, Table 1). To construct pGL3-PTEN, amplified DNA fragments were digested with Kpn I and Bgl II, and subcloned into the pGL3-basic vector (Promega, Madison, WI, USA). Before transfection, A549 cells were plated in 24-well plates at a density of 50,000 cells/well and grown overnight. Cells were co-transfected with 0.8 μg/well of the pGL3-PTEN construct and 0.5 ng/well Renilla luciferase control plasmid (PRL-SV40) by Lipofectmine 2000 (Invitrogen, Shanghai, China). After 24 h, cells were treated with dexamethasone for another 24 h. Luciferase activity was assayed by using a dual-luciferase reporter assay system (Promega) on a luminometer (GloMax 20/20, Promega).

### Trichostatin A (TSA) and anacardic acid treatment

To analyze the relationship among dexamethasone, histone acetylation and PTEN expression, the A549 cell line was allowed to grow overnight to 70% confluency in 6 well plates. The next day, TSA (Sigma, Shanghai, China) were added directly to the cells to a final concentration of 1 μmol/L. An equivalent volume of vehicle (DMSO) was added to the control. After a 24 h incubation, cells were harvested and total RNA was prepared as described for RT-PCR analysis. In anacardic acid (Sigma) experiments, cells were treated with dexamethasone (10^-5 ^M) alone or dexamethasone (10^-5 ^M) plus anacardic acid (20 μmol/L) for 24 h.

### Statistical analysis

Results were expressed as the mean ± SD. Variance analysis was used for statistical comparisons between groups by Student's t-test. Statistical significance was set at p < 0.05.

## Results

### Restoration of PTEN expression in OVA-treated mice with dexamethasone

**Figure 1:**
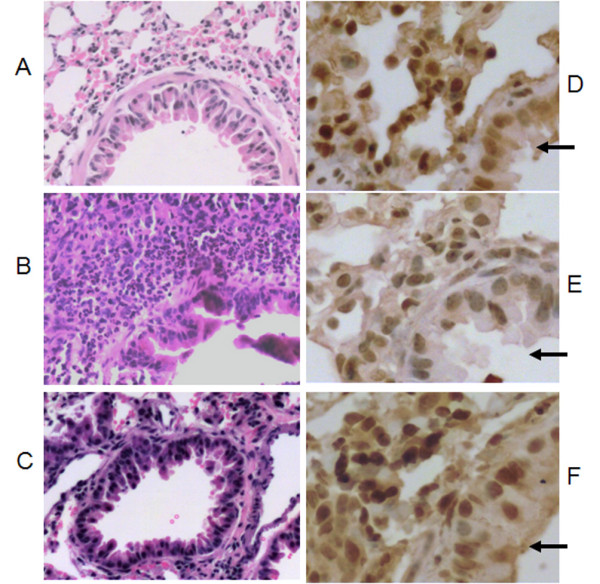
**Histological evidence of airway inflammation (A-C) and PTEN expression determined by immunohistochemical staining(D-F) and the arrows pointing to the epithelia cells**. (A and D): Lung tissue from mouse sensitized with saline. (B and E): Lung tissue from mouse after sensitization with OVA. (C and F): Lung tissue from mouse after sensitization with OVA, and treatment with dexamethasone.

**Figure 2:**
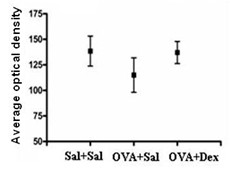
**Immunohistochemistry analysis of PTEN protein by average optical density (mean ± SD)**. Bars, 25 μm.

The lung tissues in the dexamethasone treated groups had a marked inhibition of OVA-induced inflammation including lower infiltration of eosinophils and lymphocyte, decreased of airway smooth muscle thickening and collagen deposition (Figure 1A-C), which are consistent with published data [20,21]. Immunohistochemistry was used to detect the PTEN protein expression level in lung tissue. PTEN was expressed mainly in epithelial layers around the bronchioles (Figure 1D). This immunoreactive PTEN protein was under-expressed in the OVA-treated group compared with the saline control groups (Figure 1E). However, when mice in the OVA-treated group were treated with dexamethasone, the PTEN expression in lung tissues was restored (Figure 1F). The average optical density was measured also (Figure 2). The OVA-treated group showed a significantly lower density compared with the saline group (*p *= 0.007) and OVA plus dexamethasone (*p *= 0.008).

### Dexamethasone promotes the expression of PTEN by stimulating PTEN transcription

**Figure 3:**
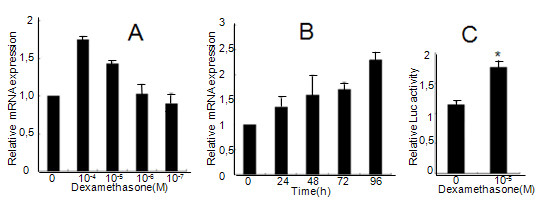
**Effect of dexamethasone on PTEN regulation in A549 cells**. A representative of three independent experiments is shown. (A): A549 cells were treated with dexamethasone at the indicated concentrations for 24 h. (B): A549 cells were treated with dexamethasone (10^-5 ^M) for 24 h, 48 h, 72 h and 96 h. The PTEN mRNA level was measured by quantitative real-time PCR. (C): A549 cells were transfected with the PTEN promoter luciferase plasmid for 24 h and treated with 10^-5 ^M dexamethasone for another 24 h. The luciferase levels were obtained from three experiments performed in duplicate. *p < 0.05 vs control group (p = 0.0003).

To further confirm the role of dexamethasone in PTEN expression, human A549 lung epithelial cells were treated with dexamethasone at the concentration 10^-5 ^M-10^-8 ^M for 24 h, or at the concentration 10^-5 ^M and harvested at 24, 48, 72, 96 h. The expression of PTEN mRNA was analyzed by real-time PCR. As shown in Figure 3A and 3B, dexamethasone treatment increased PTEN mRNA expression in a dose- and time-dependent manner, indicating that the effects of dexamethasone on PTEN expression might have occurred at the transcriptional level. To confirm this hypothesis, the PTEN promoter was cloned and constructed into the pGL3 luciferase plasmid, as described in the Methods section. We found that dexamethasone (10^-5 ^M) treatment significantly increased the PTEN promoter activity (Figure 3C), indicating that dexamethasone promoted the expression of PTEN by stimulating PTEN transcription.

### The effect of acetylation of histone on the regulation of PTEN expression

**Figure 4:**
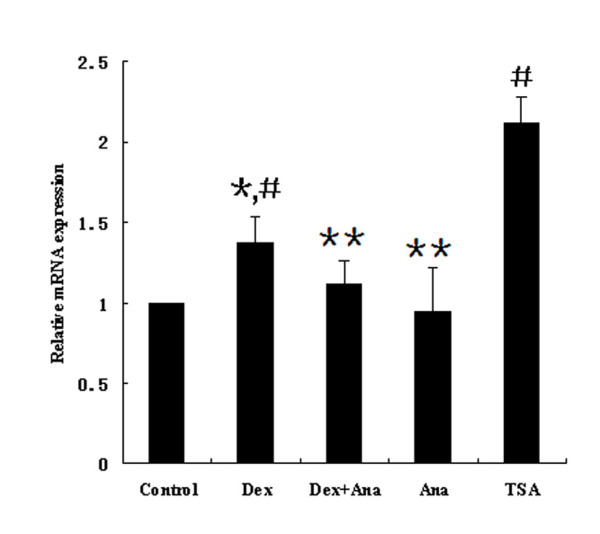
**Effect of anacardic acid, dexamethasone and TSA on PTEN expression in A549 cells**. Cells were incubated with dexamethasone (10^-5 ^M), the HAT inhibitor anacardic acid (20 μmol/L) plus dexamethasone (10^-5 ^M), anacardic acid (20 μmol/L) or TSA(1 μmol/L) for 24 h. The PTEN mRNA level was measured by quantitative real-time PCR. A representative of three separate experiments is shown. ^#^*p *= 0.006 vs control group; *^#^*p *= 0.0469 vs Dex+Ana group; **p > 0.05 vs control group.

As histone acetylation is one of the important mechanisms for the effect of glucocorticoids [22], we hypothesized that the regulation of PTEN expression by dexamethasone might involve histone acetylation. We treated A549 cells first with TSA, and confirmed that histone deacetylase inhibition was associated with the up-regulation of PTEN transcription (*p *= 0.006) (Figure 4), an observation that was consistent with a previous report [23]. We then treated A549 cells with dexamethasone (10^-5 ^M) plus the histone acetylase inhibitor anacardic acid (20 μmol/L) for 24 h. We extracted the total RNA and analyzed it by real-time PCR. We found that dexamethasone (10^-5 ^M) alone increased PTEN mRNA expression, whereas treatment with anacardic acid attenuated the dexamethasone-induced up-regulation of PTEN mRNA (Figure 4), indicating that histone acetylation inhibition is involved in the dexamethasone-induced PTEN expression.

## Discussion

OVA-induced asthma mice model is widely used for study of human asthma because of resemblance pathology and pathophysiology. Based on this model, we confirmed that PTEN proteins were under-expressed in mice with OVA-induced asthma. We also found that treatment of these mice with dexamethasone resulted in the restoration of PTEN expression. *In vitro *studies using human lung epithelial cell A549 revealed that dexamethasone was able to increase both PTEN promoter activity and gene expression. Data from all these assays together suggest that the effect of glucocorticoids on asthma may partly pass through the PTEN signaling pathway, and that PTEN is a new target gene involved in the response to dexamethasone. Although PTEN is a highly conserved gene with more than 80% identity in the promoter region between *Homo sapiens *and *Mus musculus*, more valuable data may be derived from humans. Thus, further studies in asthma patients is necessary.

The mechanisms of glucocorticoids in anti-inflammatory treatment for asthma have been investigated extensively. These studies were focused on different targets of airway or different gene expression, and had provided some answers regarding the mechanisms. The target cells studied for glucocorticoid action were mainly airway epithelial cells [24], airway smooth muscle cells [25-29], and inflammatory cells, such as mast cells [30] and monocytes [31,32]. All these effects could also be divided into genomic and non-genomic mechanisms depending on gene expression [7]. More studies will continue to draw a full picture of the mechanisms of glucocorticoids in asthma therapy. Here a new mechanism is proposed: glucocorticoids up-regulate PTEN transcription, and PTEN, in turn, inhibits inflammation.

As described above, PTEN maybe a target for asthma treatment. Regulation of PTEN expression is a key for this therapy. PTEN regulation has been the subject of many studies [33-35]. Recent studies revealed that simvastatin, pravastatin, fluvastatin, dietary exposure to the soy isoflavone genistein (GEN) and phytoestrogens induce PTEN expression in mammary epithelial cells *in vivo *and *in vitro *[36,37]. Trichostatin A (TSA) could up-regulate PTEN transcription [23]. The venom of the scorpion *Buthus martensii *Karsch upregulates the expression of PTEN, accompanied by decreased levels of Akt and Bad phosphorylation [38]. However, TGF-β1, estrogen, and PRL-3 could down-regulate PTEN expression [39,40]. There are few reagents that can specifically regulate PTEN expression in the airways. We believe more efforts should be made in this area.

With respect to the regulation inflammatory genes, glucocorticoids increase gene expression through alterations in chromatin structure by histone acetylation and recruitment of RNA polymerase II to the promoter site. This, in turn, results in the activation of gene transcription [41]. We have tested whether histone acetylation participates in the regulation of dexamethasone-induced PTEN transcription. As shown in Figure 3, the histone acetylase inhibitor anacardic acid inhibited dexamethasone-induced PTEN up-regulation in mRNA levels, indicating that histone acetylase inhibition is associated with transcriptional stimulation of the PTEN gene by dexamethasone. Our results are supported by the findings of Ito et al. [42] that high concentrations dexamethasone (>10^-8 ^M) produce a time- and concentration-dependent increase in histone acetylation in A549 cells, resulting in the recruitment of the activated transcription complex, and the subsequent increase in the expression of several genes.

The direct effect of glucocorticoids on transcript activation occurs through binding and activation glucocorticoid receptors (GR), which results in the translocation of glucocorticoid-receptor complexes to the nucleus and binding to glucocorticoid response elements (GREs) in the promoter region of target genes [43]. GREs are short sequences of DNA within the promoter that are able to bind glucocorticoid-receptor complexes and therefore regulate gene transcription. The typical DNA sequence of the GRE is 5'-GGTACAnnnTGTTCT-3' [44]. However, this typical response element could not be found in the 5'-upstream region of the PTEN gene. Several studies have reported several alternative GREs, in addition to the typical GRE [45-47]. These GREs have some variability at several nucleotide positions. Among them, the sequence 5'-TGTNC-3' was reported to be a pentamer GRE core sequence [47]. We screened the promoter region of PTEN (from -778 to -2141) for homology to this sequence. Two regions with the highest homology are at positions -1360 to -1364, and -1604 to -1608, both with the sequence 5'-TGTGC-3'. Further investigations are necessary to answer whether glucocorticoids increase PTEN expression by direct binding to these two putative GREs in the PTEN promoter region, or by interfering with the binding of other transcription factors.

In fact, the number of genes directly regulated by glucocorticoids was limited, whereas many genes were indirectly regulated through an interaction with other transcription factors and coactivators. Pan et al. reported that p300 could promote PTEN expression [23]. Wang et al. reported that dexamethasone treatment increased SRC-1, CBP and p300 recruited to the PEPCK gene promoter [48]. Recruitment of these transcription factors promotesd large protein complexes such as RNApolymerase II binding to the promoter region. Therefore it was very likely that these transcription factors participated in dexamethasone-induced PTEN regulation.

Here we propose a new signaling pathway of anti-inflammatory responses. Glucocorticoid up-regulates PTEN expression, which dephosphorylates the signal lipid PIP3 and down-regulates PIP3/AKT actions in turn. As main inflammatory mediators, the downstream targets are inhibited, thus, asthma could be controlled.

## Conclusion

Our study indicates that dexamethasone increases the expression of PTEN in asthmatic mice and human A549 cells. This induction results from the stimulation of PTEN transcription, and may involve the increased histone acetylation at the PTEN promoter. A new mechanism of action is proposed for the anti-inflammatory effect of glucocorticoids in asthma treatment. Specific regulation of PTEN expression in human airways may be useful for the treatment of asthma.

## List of abbreviations

GR: glucocorticoid receptors; GREs: glucocorticoid response elements; HAT: histone acetylase; OVA: ovalbumin; PI3K: phosphoinositide 3 kinase; PPARgamma: peroxisome proliferator-activated receptor gamma; PTEN: Phosphatase and tensin homolog deleted on chromosome 10; TSA: trichostatin A

## Declaration of interests

The authors declare that they have no competing interests. The authors alone are responsible for the content and writing of the paper.

## Authors' contributions

ZHN carried out the molecular studies and drafted the manuscript. JHT participated in the design of the study and carried out cell culture. ZYC carried out the assays of reporter construct. WY performed the statistical analysis. LZ carried out immunohistochemistry. QGC and LZ carried out animal studies. XBW conceived the study, and participated in its design and coordination and helped to draft the manuscript. All authors read and approved the final manuscript.

## Authors' information

Wang XB, Ph.D., M.D., Director of the Department of Respiratory Medicine, Putuo hospital, Shanghai university of Chinese Medicine, The Ph.D. was conferred by Karolinska Institute, Sweden in 2003. The research area is mainly focused on immunol regulation and 17 articles have been published in peer-reviewed journals.
